# Proton Pump Inhibitors Were Associated With Reduced Pseudocysts in Acute Pancreatitis: A Multicenter Cohort Study

**DOI:** 10.3389/fphar.2021.772975

**Published:** 2021-12-14

**Authors:** Shengyu Zhang, Ziying Han, Yuelun Zhang, Xiaomao Gao, Shicheng Zheng, Ruifeng Wang, Dong Wu

**Affiliations:** ^1^ State Key Laboratory of Complex Severe and Rare Diseases, Department of Gastroenterology, Peking Union Medical College Hospital, Chinese Academy of Medical Sciences and Peking Union Medical College, Beijing, China; ^2^ Medical Research Center, Peking Union Medical College Hospital, Chinese Academy of Medical Sciences and Peking Union Medical College, Beijing, China; ^3^ Clinical Epidemiology Unit, International Clinical Epidemiology Network, Beijing, China; ^4^ Department of Gastroenterology, The Sixth Hospital of Beijing, Beijing, China; ^5^ Department of Gastroenterology, West China Longquan Hospital Sichuan University, Chengdu, China; ^6^ Department of Gastroenterology, The Fourth Affiliated Hospital of Harbin Medical University, Harbin, China

**Keywords:** proton pump inhibitor, acute pancreatitis, propensity score matching, pancreatic pseudocyst, outcome

## Abstract

**Background:** Acute pancreatitis (AP) is a systemic inflammatory disorder with a wide spectrum of clinical symptoms that can range from mild to severe. Previous preclinical study results suggest that proton pump inhibitors (PPIs) can inhibit exocrine pancreatic secretion and exert anti-inflammatory properties, which might in turn improve the outcome of AP.

**Aim:** We conducted this multicenter, retrospective cohort study to investigate the potential effects of PPIs on the mortality, and total duration of hospital stay and local complication occurrence of patients with AP.

**Methods:** A total of 858 patients with AP were included. All patients presented to the hospital within 48 h of symptom onset and were divided into the following two groups: patients who were treated with PPIs (*n* = 684) and those not treated with PPIs (*n* = 174). We used propensity score matching (PSM) analysis to reduce confounding bias before comparing the outcomes between the two groups.

**Results:** Before PSM analysis, there were significant differences in a number of parameters between the two groups, including age, sex, hematocrit, blood urea nitrogen, peritonitis signs, Ranson’s score, and Acute Physiology Chronic Health Evaluation II score and organ failure occurrence. Before PSM, the PPIs group had a higher rate of mortality than the control group [RR = 1.065; 95% confidence ratio (CI) 1.045–1.086; *p* = 0.001]. After PSM, there was no significant difference in mortality (RR = 1.009; 95% CI, 0.999–1.019; *p* = 0.554) or total hospital stay (*p* = 0.856), although the PPIs group had a lower occurrence of pancreatic pseudocyst (RR = 0.416; 95% CI 0.221–0.780; *p* = 0.005).

**Conclusion:** This study showed that PPIs therapy was not associated with reduced mortality or total hospital stay, but was associated with a reduction in the occurrence of pseudocysts in patients with acute pancreatitis.

## Introduction

Acute pancreatitis (AP) is a major gastrointestinal disorder that typically requires acute hospitalization and can present a physical, emotional and socioeconomic burden worldwide (Lankisch et al., 2015). AP has a wide spectrum of clinical characteristics that can range from a mild and self-limiting condition to a more severe form of the disease, which is characterized by systematic inflammation, and organ failure and even death (Cuthbertson and Christophi, 2006; Landahl et al., 2015; Lankisch et al., 2015). Despite decades of investigation and clinical trials being performed, no specific therapeutic option has been proven to be fully effective for AP (Cavallini and Frulloni, 2001; Tenner et al., 2013; Yasunaga et al., 2013; Crockett et al., 2018). Therefore, the current practice guidelines for AP recommend supportive measures, such as intravenous fluid resuscitation, analgesia, and enteral nutrition (Tenner et al., 2013; Working Group, 2013; Greenberg et al., 2016).

Despite the lack of conclusive evidence, proton pump inhibitors (PPIs) have been empirically used as the standard therapeutic method for AP in many countries worldwide, including Japan, Korea, China, Italy, and those in Eastern Europe (Pezzilli et al., 2007; Hajjar et al., 2012; Xia et al., 2012; Yoo et al., 2012; Gorsky et al., 2015; Murata et al., 2015). PPIs act by effectively reducing the quantity of intraluminal acid in the stomach and duodenum, mechanistically by blocking the common terminal pathway for acid secretion, which lies at the H^+^-K^+^-adenosine triphosphatase (ATPase) (Robinson, 2004). H^+^-K^+^-ATPase are not only expressed in gastric parietal cells but also in the exocrine pancreas, where they drive two opposite processes, namely acid and base production, in the stomach and the pancreas, and respectively (Novak et al., 2013). It was previously observed that inhibition of H^+^-K^+^-ATPase by PPIs lead to a marked reduction of pancreatic secretion of digestive enzymes and bicarbonate (Wang et al., 2015). Indeed, H^+^-K^+^-ATPase is essential for the secretion of bicarbonates and has been associated with the premature activation of zymogen enzymes in pancreatic acinar cells (Kukor et al., 2002; Novak et al., 2011), which is considered to be an important trigger for the initiation of AP (Gukovsky et al., 2012; Lankisch et al., 2015). Furthermore, previous experimental studies have shown that PPIs can exert anti-inflammatory effects by inhibiting leukocyte migration *in vitro* (Handa et al., 2006; Martins de Oliveira et al., 2007). PPIs can also scavenge hydroxyl radicals *in vitro*, which might improve the outcome of AP (Simon et al., 2006). In previous studies on animal models, pantoprazole has been shown to attenuate AP by reducing the expression of inflammatory markers, and adhesive factors such as CD31 (Hackert et al., 2010). From the body of evidence aforementioned, it would seem plausible to evaluate if PPIs should be used more frequently to treat patients with AP.

However, the clinical evidence regarding the therapeutic efficacy of PPIs for AP is scarce, such that results reported to date are inconclusive. In a previous case report, lansoprazole has been used successfully to treat and prevent recurrent AP that was induced by chemotherapy in children with acute lymphoblastic leukemia/lymphoma (Fettah et al., 2014). By contrast, a retrospective study conducted in Japan using the national administrative database revealed that the use of PPIs did not reduce the mortality in patients with severe acute pancreatitis (Murata et al., 2015), in which data of other outcomes, including local complications and the length of hospital stay, and were not reported. At present, to the best of our knowledge, there has only been one prospective study that demonstrated that pantoprazole did not alter the clinical course of AP (Yoo et al., 2012), although a limited sample size (40 in total), and the heterogeneity among the enrolled patients render these results ambiguous (Yoo et al., 2012). Demcsak *et al* also revealed that PPIs treatment conferred no benefits on the outcome of AP, but this study only analyzed the mortality rate, and the gastrointestinal bleeding occurrence as outcomes (Demcsák et al., 2020).

Therefore, we conducted this study to investigate the potential effect of PPIs on the mortality, in-hospital stay and the rate of local complication occurrence of patients with AP from a number of tertiary teaching hospitals in China.

## Materials and Methods

### Patients

We conducted this comparative effectiveness research based on a prospectively maintained dataset in Peking Union Medical College Hospital (Beijing, China), The Fourth Affiliated Hospital of Harbin Medical University (Harbin, China), and the Longquanyi Branch of West China Hospital (Chengdu, China). Approval from Peking Union Medical College Hospital Ethics Committee was obtained prior to this study (approval no. S-K919) and informed consent was collected from patients enrolled for data collection and manuscript publication.

All patients with the first episode of AP were identified, who presented to the hospital within 48 h of symptom onset between January 2004 and December 2019. Diagnosis of AP was made if two of the following three criteria were fulfilled: 1) Abdominal pain characteristic of AP; 2) serum amylase/lipase values of more than three times the upper limit of normal; and 3) imaging evidence of AP (Tenner et al., 2013). Exclusion criteria included those younger than 18 years old, those with a past history of PPIs use, those with underlying chronic pancreatitis and those who departed from the hospital before a full investigation, and/or treatment of AP was completed. The basic treatment regimens included early fluid resuscitation, analgesia and nutritional support (parenteral or enteral) according to practice guidelines (Tenner et al., 2013; Crockett et al., 2018).

### Management of Acute Pancreatitis

The basic treatment regimens included early fluid resuscitation, analgesia and nutritional support (parenteral or enteral) according to the American Gastroenterological Association (ACG) guidelines. Local complications and organ failure were treated symptomatically and timely with continuous close monitoring (Tenner et al., 2013; Crockett et al., 2018). Antibiotics were provided for patients with infected necrosis but not for sterile necrosis. No traditional Chinese Medicine was used in these patients.

### Study Variables

All data were collected from an electronic medical record database of patients who were diagnosed with AP as their primary condition. For all enrolled patients, the following parameters were recorded within the first examination, which took place within 24 h of admission: 1) age; 2) sex; 3) time between the onset of symptoms and admission; 4) etiology; 5) hematocrit (Hct); 6) blood urea nitrogen (BUN); and 7) signs of peritonitis. Ranson’s score, which can predict the severity of AP, and was assessed within 48 h of admission by doctors (Harshit Kumar and Singh Griwan, 2018). In addition, the Acute Physiology Chronic Health Evaluation II (APACHE II) score, organ failure (OF), and PPIs use were evaluated before each of the patients’ discharge.

PPIs use was defined as receiving intravenous omeprazole, pantoprazole, or/and esomeprazole at least once during hospitalization (Murata et al., 2015). Etiology was divided into four types (Wang et al., 2009). Alcohol-related pancreatitis was defined as a history of excessive intake of alcohol prior to the onset of symptoms. Hypertriglyceridemia-associated pancreatitis was considered when the serum triglyceride level at admission was more than 11.3 mmol/L (1,000 mg/dl) and no signs of other risk factors (Mosztbacher et al., 2020). Gallstone-related pancreatitis was diagnosed when gallstones could be visualized via radiological examination and/or an elevation in the bilirubin value, which is frequently observed with a concomitant rise in alanine aminotransferase and alkaline phosphatase levels, and was present at the time of admission to the hospital. Other identified causes of AP were trauma, hypercalcemia, malignancy, infection, and endoscopic retrograde cholangiopancreatography (Wang et al., 2009). OF was defined as a score of 2 or more for one of these organ systems, including respiratory, renal, and cardiovascular system, by using the modified Marshall score (Banks et al., 2013).

Patients were also categorized into mild, moderately severe and severe AP, which were defined using the revised Atlanta classification 2012 (Banks et al., 2013): 1) Mild AP (MAP):AP without OF or local complications (LCs); 2) moderately severe AP (MSAP): AP with OF that resolves within 48 h (transient OF) and/or AP with LCs; and 3) severe AP (SAP), which is AP with persistent OF (more than 48 h).

### Outcome

The primary outcome of the present study is in-hospital mortality. Secondary outcomes include total hospital stay and local complications during hospitalization. Potential LCs were examined via computed tomography (CT) before discharge. Pancreatic pseudocyst (PP) was defined as an encapsulated collection with a well-defined wall without necrotic debris being observed more than 4 weeks after onset. Walled-off necrosis (WON) was defined as a mature encapsulated collection of pancreatic with/without peripancreatic necrosis that has developed a well-defined wall, 4 weeks after onset of necrotizing pancreatitis. Infected pancreatic necrosis (IPN) was defined by at least one of the following findings: Presence of gas bubbles in the pancreas and/or peripancreatic tissue following CT examination, a positive result in smear staining or culture of samples obtained by image-guided fine needle aspiration, and or during the first intervention (either by drainage or surgery) (Banks et al., 2013).

### Statistical Analysis

To correct for the unbalanced baseline characteristics between the two groups, we used propensity score matching (PSM) to adjust for the confounding effects (Baek et al., 2015). PSM used the nearest-neighbor strategy with a caliper width of 0.2. In this study, PSM was estimated according to the variables including age, sex, time between the onset of symptoms and admission, etiology, Hct, BUN, peritonitis signs, Ranson’s score, APACHE II score, and organ failure. To increase the utility of data, the matching ratio was 1:2 depending on the distribution of the two groups, and the success rate of matching.

The balance of the matched cohort was evaluated using absolute standardized differences (ASD) (Bangalore et al., 2015). Differences in baseline characteristics between the two groups was considered to be small when the ASD was calculated to be less than 0.1.

In the matched cohorts, comparison of the in-hospital mortality rate was performed using the χ^2^ test or Fisher’s exact test. Comparison of total hospital stay was performed using the nonparametric Mann-Whitney test, whilst the rate of LCs was also compared using the χ2 test or Fisher’s exact test. The risk ratio (RR) and 95% confidence intervals (CI) were also calculated.

We also conducted two sensitivity analyses. In total, 348 samples were removed during the PSM analysis. To exclude the effects of reduced statistical power in PSM analysis, we used bivariate logistic regression analysis to assess the relationship between treatment with PPIs, and the major outcomes before PSM. After PSM, potential imbalance in the rate of acute respiratory distress syndrome (ARDS) between the two groups still existed. Therefore, to eliminate any residual confounding factors, and we conducted subgroup analyzes according to ARDS after PSM. We used the Fisher’s exact test to compare the mortality rate between the PPIs and the non-PPIs groups.

Statistical analyses in this study were performed using SPSS 26.0 software for Windows (IBM Corp., Armonk, NY, and United States ) and R version 3.5.0 (R Foundation for Statistical Computing, Vienna, and Austria). A two-tailed *p*-value of less than 0.05 was considered to indicate a statistically significant difference.

## Results

### Population Characteristics

Information of 1,102 patients was collected from medical record library according to inclusion criteria, and 244 patients were excluded for incomplete data. A total of 858 patients were enrolled, which included 500 patients with MAP, 204 with MSAP, and 154 with SAP. Patients were divided into the following two groups: those who had been treated with PPIs [*n* = 684; 369 (53.9% with MAP), 169 (24.7% with MSAP), and 146 (21.3% with SAP)] during hospitalization; and those not treated with PPIs [*n* = 174; 131 (75.3% with MAP), 35 (20.1% with MSAP), and 8 (4.6% with SAP)]. The clinical characteristics of both groups are shown in Table 1. Prior to PSM, patients tended to be younger (52.65 ± 17.49 vs 55.40 ± 17.03 years; ASD = 0.162) in the PPIs group, and which also contained a higher proportion of men (64.62 vs 52.3%; ASD = 0.246). Gallstone remains to be the leading cause of AP in both PPIs and non-PPIs groups (52.8 vs 56.3%). There were significant differences between the PPIs group and the non-PPIs group in a number of parameters, including age, sex, Hct, BUN, peritonitis signs, Ranson’s score, APACHE II score, and OF occurrence, which are significantly higher in the PPIs group.

**TABLE 1:** Comparison of clinical characteristics before and after matching.

Characteristics	Before Matching			After Matching		
	PPIs group (*N* = 684)	No PPIs group (*N* = 174)	Absolute standardized difference	PPIs group (*N* = 336)	No PPIs group (*N* = 174)	Absolute standardized difference
Age in years	52.65 ± 17.49	55.40 ± 17.03	0.162	56.59 ± 17.17	55.40 ± 17.03	0.006
Male, *n* (%)	442 (64.62)	91 (52.3)	0.246	183 (54.5)	91 (52.3)	0.046
Duration of symptoms at admission in hours (median, IQR)	23.06 ± 32.97 (12.00,18.00)	27.68 ± 47.79 (12.00,18.00)	0.097	22.96 ± 32.55 (12.00,19.00)	27.68 ± 47.79 (12.00,18.00)	0.076
Etiology			0.056			0.006
Gallstone, n (%)	361 (52.8)	98 (56.3)		195 (58.0)	98 (56.3)	
Lipogenic, n (%)	81 (11.84)	11 (6.32)		22 (6.5)	11 (6.32)	
Alcohol, n (%)	51 (7.46)	6 (3.44)		19 (5.7)	6 (3.44)	
Others, n (%)	190 (27.78)	59 (33.9)		100 (29.8)	59 (33.9)	
Hematocrit at admission	41.56 ± 6.45	40.30 ± 5.34	0.236	40.53 ± 5.45	40.30 ± 5.34	0.031
blood urea nitrogen at admission	5.99 ± 4.31	5.00 ± 2.06	0.481	5.07 ± 2.47	5.00 ± 2.06	0.032
Peritonitis signs, n (%)	86 (12.57)	4 (2.3)	0.684	5 (1.49)	4 (2.3)	0.076
Ranson’s score	1.90 ± 1.67	1.09 ± 1.02	0.796	1.18 ± 1.15	1.09 ± 1.02	0.012
APACHE II score	6.34 ± 5.22	4.38 ± 2.89	0.679	4.61 ± 3.45	4.38 ± 2.89	0.030
Organ failure, n (%)						
Acute respiratory distress syndrome, n (%)	114 (16.7)	5 (2.87)	0.823	14 (4.17)	5 (2.87)	0.103
Acute renal failure, n (%)	55 (8.04)	3 (1.72)	0.484	5 (1.49)	3 (1.72)	<0.001
Cardiovascular collapse, n (%)	28 (4.1)	1 (0.57)	0.246	4 (1.19)	1 (0.57)	0.038
Multiple organ failure, n (%)	45 (6.58)	1 (0.57)	0.792	3 (0.89)	1 (0.57)	0.042

### Logistic Regression Analysis

Before PSM, there was a significance between the two groups in terms of in-hospital mortality (*p* = 0.001; RR = 1.065; 95% CI, 1.045–1.086; Table 2). However, significant effects induced by PPIs on mortality was not found by logistic regression analysis before PSM.

**TABLE 2:** Comparison of outcomes between PPIs group and non-PPIs group.

Outcome	PPIs group	No PPIs group	RR (95%CI)	*p*-value
Primary outcome: In-hospital mortality, n (%)				
Crude	42/684 (6.14)	0/174 (0.00)	1.065 (1.045–1.086)	0.001
PSM adjusted	3/336 (0.89)	0/174 (0.00)	1.009 (0.999–1.019)	0.554
Secondary outcome (PSM adjusted)				
Local complication				
Pancreatic pseudocyst, n (%)	20/336 (5.95)	23/174 (13.2)	0.416 (0.221–0.780)	0.005
Walled-off necrosis, n (%)	11/336 (3.27)	3/174 (1.72)	1.929 (0.531–7.008)	0.466
Infected pancreatic necrosis, n (%)	9/336 (2.68)	4/174 (2.29)	1.170 (0.355–3.854)	1.000
Total hospital stay in days (median, IQR)	24.06 ± 20.63 (19.00,15.00)	26.59 ± 25.12 (18.00,19.00)		0.856

### Propensity Score Matched Analysis

After PSM, there were 336 patients in PPIs group and 174 patients in non-PPIs group. The characteristics of two groups were balanced basically, with no significant difference except for ARDS (4.17 vs 2.87; ASD = 0.103) (Table 1).

After PSM, there was no significance between the two groups with regards to in-hospital mortality (RR = 1.009; 95% CI, 0.999–1.019; *p* = 0.554; Table 2). For secondary outcomes, only the difference in the incidence of PP was significant (RR = 0.416; 95% CI, 0.221–0.780; *p* = 0.005) after PSM, but there was no significance in the difference between the two groups in total hospital stay (*p* = 0.856), WON (RR = 1.929; 95% CI, 0.531–7.008; *p* = 0.466), and IPN (RR = 1.170; 95% CI 0.355–3.854; *p* = 1.000). Results from the sensitive analysis are consistent with the results aforementioned, which found no significant differences after the subdivision of patients according to ARDS.

## Discussion

Our study was based on a real-world dataset, which focused on the mortality rate, hospital stay, and occurrence of local complications. It revealed that although PPIs were commonly used in patients with AP, they were not associated with improved prognosis. However, PPIs treatment was found to be associated with decreased risks of PP occurrence.

PPIs are potent inhibitors of gastric acid production and exocrine pancreatic secretion (Robinson, 2004; Novak et al., 2013). Currently available evidence support the notion that H^+^/K^+^-ATPases expressed in the pancreatic ducts resemble those expressed in the gastric glands, but function in the opposite manner by expelling H^+^ into the vasculature whilst reserving HCO^3−^ for luminal transport in the process (Novak et al., 2013). Previous experimental studies have demonstrated that gastric HKα1 and HKβ subunits (ATP4A; ATP4B) and non-gastric HKα2 subunits (ATP12A) of the H^+^/K^+^-ATPase machinery are expressed in human pancreatic ducts (Wang et al., 2015; Tozzi et al., 2020). Given the functional and structural similarities reported between gastric and pancreatic H^+^/K^+^-ATPases, it is therefore not surprising that PPIs can inhibit exocrine pancreatic secretion by suppressing ductal proton pumps (Novak et al., 2011; Wang et al., 2015). Furthermore, PPIs can mitigate the proinflammatory cascade and reduce oxidative stress, and which are key pathophysiological events in AP (Handa et al., 2006; Simon et al., 2006; Martins de Oliveira et al., 2007). These observations may explain why PPIs were associated with PP occurrence in this study, which appears to benefit patients with AP.

Although PPIs were found to be associated with a decreased risk of PP, they were not associated with the occurrence of WON, IPN, and total hospital stay or mortality in our study. In previous studies, PPIs have been reported to increase the risk of necrosis and infection (Min et al., 2019; Powers et al., 2019). Drainage is the main option for pancreatic necrosis, but a recent study revealed that reducing PPIs use can decrease the number of endoscopic procedures for treating WON (Powers et al., 2019). Peripancreatic infection can be caused by intestinal bacteria translocation after compromised gastrointestinal barrier function (Demcsak et al., 2020). Long-term PPIs use may damage the gut barrier and alter the gut microbiome, thereby elevating the risk of gastrointestinal (GI) infections. Supporting this, a previous study reported that PPIs use can increase the risk of cholangitis (Min et al., 2019). In addition, PPIs may cause alterations in the gut microbiome, and which may facilitate the development of systemic and local infections prior to severe acute pancreatitis (Clooney et al., 2016; Ma et al., 2020). The total period of hospital stay and mortality rate will likely increase for patients with intraperitoneal infection or pancreatic necrosis (Rashid et al., 2019). Although antibiotics were shown to confer no benefits in preventing necrosis or reducing mortality (Párniczky et al., 2019), unnecessary antibiotic treatment is widely applied in patients with AP, and which may control GI infections and can influence analysis results (Villatoro et al., 2010; Demcsák et al., 2020).

As mentioned above, study results of PPIs efficacy in AP patients are conflicted. This may be because AP can be considered to be a systemic disease with potentially far-reaching complications, including shock, and renal insufficiency and respiratory failure (Lankisch et al., 2015). Organ failure is not only secondary to local complication simply and may precede necrosis although the mechanism is still unknown (Garg and Singh, 2019). Therefore, medications targeting local pancreatic inflammation instead of the systemic cytokine storm may not mitigate the global complications resulting from AP. Therefore, although PPIs may contribute to controlling local pancreatic secretion and inflammation, they may not be as effective in attenuating AP-associated systematic inflammatory responses, and multiple organ failures or mortality. This hypothesis is consistent with the results from the present study. In addition, the present study showed that OF and other risk factors of poor prognosis were significantly more common in patients who were treated with PPIs than in those who did not before PSM. It was most likely due to the fact that physicians are more inclined to prescribe PPIs for severe cases of AP due to the presumed benefits and for preventing stress ulcers. A similar phenomenon was also found in a previous study performed by Murata *et al*, where patients with more severe AP conditions were more likely to receive PPIs (Murata et al., 2015). These factors may influence the effect of PPIs on the outcome of AP. These findings also encourage the use of PSM for minimizing the effect of bias.

Another aim of PPIs use is to prevent or treat stress-related GI mucosal lesions and bleeding in patients with AP. However, the occurrence of GI bleeding is uncommon in AP, and especially among patients with non-severe condition. A previous cohort study showed that GI bleeding only occurred in 2.1% of patients with MAP (Demcsák et al., 2020). As the predominant part of enrolled patients were with MAP in this study, the potential effect of PPIs on preventing stress ulcer, and GI bleeding may be difficult to evaluate at present. Therefore, the role of PPIs in clinical treatment for patients with AP require further study.

A number of limitations in the present study remain. Only omeprazole, pantoprazole and esomeprazole were administered intravenously in the present study due to the limited selection of intravenous PPIs available in these tertiary hospitals. This excluded the possibility of evaluating the effect of other PPIs. We defined PPIs users as those who received intravenous PPIs treatment at least once during hospitalization, regardless of the dosage, and duration of PPIs used. Therefore, we were unable to determine the effect of oral administration or to establish a dose-effect relationship in the present study. In addition, enteral nutrition is a crucial factor for prognosis determination (Song et al., 2018). The duration and regimens of enteral nutrition was not included in the present analysis. However, the application of enteral nutrition for all patients followed the ACG guidelines (Tenner et al., 2013; Crockett et al., 2018). Moreover, to balance the therapy tendency of PPIs, we excluded a substantial number of cases during PSM analysis, and especially patients with critical disease. This may have led to a reduction in sample size and the mortality rate, thereby decreasing the statistical power. Lastly, there are methodological limitations of a retrospective study. Although we identified a possible role of PPIs to reduce pseudocyst and suggested related mechanisms, it’s difficult to establish a cause-and-effect relationship. The true efficacy of PPIs to treat AP calls for prospective randomized controlled studies.

In conclusion, the present study showed that PPIs treatment was not associated with improving mortality or total hospital stay, and but it was related to a reduction in the occurrence of pancreatic pseudocysts in patients with acute pancreatitis. Considering the potentially increased risk of peripancreatic infection, further prospective studies are required for clarifying the clinical application of PPIs.

## Data Availability

The raw data supporting the conclusion of this article will be made available by the authors, without undue reservation.
